# Prisoners Treated for Hepatitis C with Protease Inhibitor, New York, USA, 2012

**DOI:** 10.3201/eid2101.141025

**Published:** 2015-01

**Authors:** Harish Moorjani, Carl Koenigsmann, Min Jung Kim, Anne C. Spaulding

**Affiliations:** New York Medical College, Valhalla, New York, USA (H. Moorjani);; New York State Department of Corrections and Community Supervision, Albany, New York, USA (C. Koenigsmann);; Emory University, Atlanta, Georgia, USA (M.J. Kim, A.C. Spaulding)

**Keywords:** Hepatitis C, viruses, prisons, direct acting agents

**To the Editor**: Globally, epidemics of infection with hepatitis C virus (HCV) tend to be concentrated in correctional facilities, according to a review of worldwide literature during 1990–2012 (*1*,*2*). Nearly 1 in 3 persons infected with HCV in the United States spends at least part of each year in either a prison or jail (*3*). Proficiency by correctional health facilities in hepatitis C treatment by using the most effective agents may mitigate the predicted burden of end-stage liver disease and hepatocellular cancer in the coming years. The first wave of direct-acting agents against HCV had substantial side effects. Nonetheless, the New York Department of Corrections and Community Supervision (NYDOCCS) systematically approached the challenge of using these agents, and their experience serves as a lesson that prison medical services can overcome substantial barriers to care.

In 2011, NYDOCCS piloted a hepatitis C treatment program for HCV genotypes 1a and 1b, comprised of telaprevir, pegylated interferon, and ribavirin. Patients underwent extensive mandatory pretreatment screening for mental health issues, pregnancy, and cirrhosis by using the FibroSURE assay (LabCorp, Burlington, NC, USA) and Doppler sonogram of the portal vein. Eligibility requirements were a negative HIV test result, and liver fibrosis assessed either by liver biopsy or noninvasively, at METAVIR stage 2 or 3, or 4 (reference range 0–6) (*4*) with compensation. A patient in stage 1 was eligible if the patient had poor prognostic factors. An infectious disease consultant saw every patient; each patient received mandatory patient education. Mandatory conditions for patients participating in the program were to sign a consent form agreeing to the medication, participate in laboratory monitoring, and attend primary or specialty clinic follow-up appointments; 3 refusals of any program component in any combination would result in medication discontinuation. Because referring physicians conducted the initial screening of potential participants, the exact number of patients screened cannot be determined. The most frequent factor for ineligibility was uncontrolled psychiatric disease.

Facilities were encouraged to treat <5 patients at any given time. Staff physicians, mid-level providers, nurses, pharmacists, and social workers participated in extensive training. Telaprevir was administered with a fat supplement by directly observed therapy. Patients waited in lines every 7–9 hours to receive the medications. All patients received antihistamine prophylaxis before therapy was initiated. Patients received containers of high potency topical steroid creams at the initiation of treatment with instructions to start applying it 2× daily at the first sign of a rash. The nurses questioned the patients about rash and pruritus during each weekly interferon injection appointment. The infectious disease consultant conducted follow-up visits by telemedicine, using video and audio equipment for remote monitoring and supervision of treatment.

Among the 50 patients who began therapy, 38 (76%) were naïve to any treatment, 7 (14%) were experiencing relapses, 4 (8%) had responded partially to prior treatment, and 1 (2%) had not responded to prior treatment. Ages of the patients ranged from 21 to 67 years. All 50 treated patients experienced rash, pruritus, or both. All but 1 patient (98%) experienced fatigue. Anemia was detected in 68%, yet none received erythropoietin. Rather, the infectious disease consultant aggressively reduced ribavirin doses. Because of difficult-to-access, paper-based recording of dosing, determining the mean dose of ribavirin used in each patient was not feasible. None required transfusions. Thrombocytopenia developed in 2 patients. In 64%, neuropsychiatric effects were observed. Of 50 patients, 2 (4%) dropped out of the program. As shown in the Table, 44 (88%) experienced a sustained viral response.

**Table:** Outcomes of 50 prisoners treated for hepatitis C* within the New York State Department of Corrections and Community Services, 2012–2013†

Outcome	No. (%)	Metavir staging of fibrosis	
		No. stage 1–3 (%)	No. stage 4 (%)
Disenrolled	2 (4)	1 (2)	1 (11)
Treatment failed	4 (8)	2 (5)	2 (22)
Sustained viral response	44 (88)	38 (93)	6 (67)
Early rapid viral response	34 (68)	31 (76)	3 (33)
Total	NA	41	9

*Treated with telaprevir, pegylated interferon, and ribavirin. †NA, not applicable.

By using the most effective agents available to treat hepatitis C, correctional health care systems can make a unique contribution to public health (*5*). Despite the substantial side effects of the first direct acting agents, the NYDOCCS health care personnel observed and reported a sustained viral response in a select group of patients which exceeded that seen in the drug registration trials (*6*). Recruiting a multidisciplinary health care team facilitated administration of a cumbersome regimen. Prophylactic treatment for rash and ribavirin dose reductions in 68% may have minimized dermatologic side effects. Mandatory patient education may have enhanced patient motivation to adhere to the protocol. As shown previously, telemedicine was a valuable tool in hepatitis C management of prisoner-patients (*7*). Treatment administered by the NYDOCCS treatment team cured many HCV-infected patients. Correctional health providers can manage complex therapeutic protocols for treatment of voluntary participants in the controlled environment of these facilities.

Treatment with novel agents has ushered in a new era in hepatitis C management. The cost of newer drugs is daunting (e.g., $84,000 for a 12-week course of sofosbuvir), but prison providers are starting to use this next generation of agents (*8*). Recent clinical trials with newer direct-acting agents that spare interferon have even higher rates of virologic success and fewer side effects (*9*,*10*) which should lead to even more widespread success.
